# Invasive Fungal Disease in Patients with Newly Diagnosed Acute Myeloid Leukemia

**DOI:** 10.3390/jof7090761

**Published:** 2021-09-15

**Authors:** Anastasia I. Wasylyshyn, Kathleen A. Linder, Carol A. Kauffman, Blair J. Richards, Stephen M. Maurer, Virginia M. Sheffield, Lydia Benitez Colon, Marisa H. Miceli

**Affiliations:** 1 Division of Infectious Diseases, University of Michigan Health System, Ann Arbor, MI 48109, USA; aniedz@med.umich.edu (A.I.W.); linderk@med.umich.edu (K.A.L.); stemaure@med.umich.edu (S.M.M.); vmmorris@med.umich.edu (V.M.S.); mmiceli@med.umich.edu (M.H.M.); 2 Infectious Diseases Section, VA Ann Arbor Healthcare System, Ann Arbor, MI 48105, USA; 3 Michigan Institute for Clinical & Health Research, Ann Arbor, MI 48105, USA; blairr@med.umich.edu; 4 Department of Pharmacy, University of Michigan Health System, Ann Arbor, MI 48109, USA; lbenitez@med.umich.edu

**Keywords:** invasive fungal disease, mold infection, yeast infection, acute myeloid leukemia, breakthrough fungal disease

## Abstract

This single-center retrospective study of invasive fungal disease (IFD) enrolled 251 adult patients undergoing induction chemotherapy for newly diagnosed acute myeloid leukemia (AML) from 2014–2019. Patients had primary AML (*n* = 148, 59%); antecedent myelodysplastic syndrome (*n* = 76, 30%), or secondary AML (*n* = 27, 11%). Seventy-five patients (30%) received an allogeneic hematopoietic cell transplant within the first year after induction chemotherapy. Proven/probable IFD occurred in 17 patients (7%). Twelve of the 17 (71%) were mold infections, including aspergillosis (*n* = 6), fusariosis (*n* = 3), and mucomycosis (*n* = 3). Eight breakthrough IFD (B-IFD), seven of which were due to molds, occurred in patients taking antifungal prophylaxis. Patients with proven/probable IFD had a significantly greater number of cumulative neutropenic days than those without an IFD, HR = 1.038 (95% CI 1.018–1.059), *p* = 0.0001. By cause-specific proportional hazards regression, the risk for IFD increased by 3.8% for each day of neutropenia per 100 days of follow up. Relapsed/refractory AML significantly increased the risk for IFD, HR = 7.562 (2.585–22.123), *p* = 0.0002, and Kaplan-Meier analysis showed significantly higher mortality at 1 year in patients who developed a proven/probable IFD, *p* = 0.02. IFD remains an important problem among patients with AML despite the use of antifungal prophylaxis, and development of IFD is associated with increased mortality in these patients.

## 1. Introduction

Invasive fungal disease (IFD) is a highly morbid complication in patients with hematologic malignancies, including acute myeloid leukemia (AML) [1]. Prior studies have demonstrated a benefit of mold-active antifungal agents for IFD prophylaxis in patients at high risk [2,3]. With widespread use of prophylaxis, breakthrough IFD (B-IFD) have become an increasing problem and have been reported in up to 18% of patients with AML [4,5,6,7,8,9,10]. Early studies suggested that the risk factors predisposing AML patients for B-IFD were similar to those for IFD, in general, and included underlying severe myeloid immunosuppression from leukemia, prolonged neutropenia, use of central venous catheters, mucositis from chemotherapy or from graft versus host disease (GVHD) after hematopoietic cell transplantation (HCT), and use of broad-spectrum antibiotics [10,11]. However, heterogenous populations were included and no standardized definition of B-IFD was available when those studies were performed, calling into question whether the results are generalizable [12].

The recent development of new antifungals and improved formulations of existing agents has prompted changes in antifungal prophylaxis strategies for patients with AML. Additionally, revised definitions for IFD have been developed by the European Organization of Research and Treatment of Cancer and Mycoses Study Group Education and Research Consortium (EORTC/MSGERC) [13], and a consensus definition for B-IFD has been published by the MSGERC and the European Confederation of Medical Mycology (ECMM) [14]. This new definition for B-IFD incorporates the newer antifungal agents and better defines antifungal exposure by including pharmacokinetic parameters of the prophylactic agents.

We sought to determine the effectiveness of newer strategies for the prevention of IFD in patients with AML and to better define the occurrence of B-IFD at our institution.

## 2. Materials and Methods

### 2.1. Patients and Setting

This retrospective cohort study was conducted at the University of Michigan Medical Center, a 1000 bed tertiary care center. Approval for this study was granted by the institutional review board. All adult patients at least 18 years of age who had newly diagnosed AML and who were admitted for induction chemotherapy between June 2014 and January 2019 were screened for eligibility. Patients who expired prior to initiation of induction chemotherapy and those who received umbilical cord blood HCT or more than one allogeneic HCT within 1 year after first induction chemotherapy were excluded. Study patients were followed for one year from the first day of induction chemotherapy unless death occurred before that time.

The electronic medical record was reviewed to collect data on patient demographics and comorbidities, AML status at baseline, at time of IFD diagnosis, and at the last follow up, chemotherapy regimens, HCT, graft versus host disease (GVHD), cumulative duration of neutropenia, cumulative prophylactic antifungal exposure, serum trough concentrations of prophylactic antifungals when available, outcome of IFD and B-IFD at 12 weeks from the date of diagnosis, and overall mortality at 1 year after first induction chemotherapy.

### 2.2. Definitions

AML was defined by WHO 2016 revised guidelines [15]. Induction chemotherapy regimens were determined by the primary hematology team in accordance with National Comprehensive Cancer Network guidelines and recommendations [16]. Cumulative neutropenia was defined as the total duration, in days, of an absolute neutrophil count (ANC) of <500 cells/µL for the year following first induction chemotherapy. Cumulative neutropenic days were normalized per 100 days of follow up to account for the variable follow up times for each patient. Cumulative exposure to antifungals was defined as the total number of days that prophylactic antifungal agents were given to each patient over the follow up period and was normalized per 100 days of follow up.

Proven and probable IFD, including *Pneumocystis* pneumonia, were defined by the EORTC/MSGERC 2019 revised consensus criteria [13]. The day of diagnosis of IFD was defined as the date when the diagnosis was first suspected based on clinical, radiological, and microbiological findings. Breakthrough IFD was defined based on the MSGERC/ECMM consensus definitions and included fungal infections that occurred at least 7 days after initiation of any antifungal agent or that occurred less than one day after discontinuing any antifungal agent [14].

### 2.3. Prophylaxis and Isolation Strategies

Antifungal prophylaxis for all AML patients undergoing chemotherapy was voriconazole (with therapeutic drug monitoring and a goal trough level of 1 to 5.5 µg/mL) when ANC fell to ≤1500/µL; this was continued until neutrophils were >500/µL for at least 3 consecutive days. Alternative regimens, including posaconazole, isavuconazole, fluconazole, or micafungin, were used at the discretion of the hematology team if intolerance or drug-drug interactions were present. *Pneumocystis* prophylaxis was reserved for patients receiving purine analogues; either inhaled pentamidine 300 mg monthly or double-strength trimethoprim-sulfamethoxazole (TMP-SMX) tablet three times a week was given throughout chemotherapy and for six months after the last dose of a purine analogue agent. Acyclovir was given throughout all chemotherapy cycles for antiviral prophylaxis. Bacterial prophylaxis with a fluoroquinolone was given only to patients with relapsed or refractory AML.

After allogeneic HCT, fluconazole was given for antifungal prophylaxis unless the patient met one of the following criteria: prolonged neutropenia (>21 days) after or preceding transplant; corticosteroid treatment for GVHD, engraftment syndrome or idiopathic pneumonia syndrome; calcineurin inhibitor therapy in combination with any other immunosuppressive agent; use of etanercept, alemtuzumab, or anti-thymocyte globulin for conditioning; or a history of IFD prior to transplant. Patients who met one or more of these criteria received voriconazole rather than fluconazole. Alternative regimens included posaconazole and micafungin and were used at the discretion of the transplant physician in settings of drug intolerance or drug-drug interactions. Antifungal prophylaxis continued until day +100 and until the specific risk factor for IFD was no longer present, whichever occurred later. *Pneumocystis* prophylaxis was a single-strength TMP-SMX tablet daily starting at day +30 and through day +180 or until immunosuppression was stopped, provided cell counts had recovered. Alternative agents in cases of persistent cytopenia included atovaquone or inhaled pentamidine. Acyclovir was given for antiviral prophylaxis for one year. Antibacterial prophylaxis was with a fluoroquinolone from day +1 until either engraftment or development of febrile neutropenia.

A protective environment consisting of a positive pressure room with 12 air exchanges per hour and HEPA filtration was used for allogeneic HCT patients who are in the peri-transplant period and the first 100 days post-HCT, those who have an ANC < 1000 cells/mm^3^, and those who have acute GVHD. AML patients undergoing induction therapy with an ANC < 1000/mm^3^ also are placed in this type of room.

### 2.4. Statistical Analysis

Univariable analysis accounting for competing risks (deaths) was conducted using cause-specific proportional hazards regression to evaluate the impact of cumulative days of neutropenia per 100 days of follow up, cumulative prophylactic antifungal exposure per 100 days of follow up, AML status and other patient characteristics at time of IFD. Hazard ratios and 95% confidence intervals were calculated. Further univariable analyses were performed using the Wilcoxon rank sums test. Kaplan-Meier survival analysis with a log-rank test was conducted to evaluate the impact of IFD on survival. SAS version 9.4 statistical software (SAS Inc., Cary, NC, USA) was used for all analyses.

## 3. Results

### 3.1. Patient Characteristics

Altogether, 264 patients were screened and 251 patients were entered into the study. Excluded were eight patients who died before induction therapy was accomplished, four who received a cord blood HCT, and one who received a second HCT within the year after induction therapy. Of the 251 patients, 113 were women (45%), and the average age was 62 ± 14 years (Table 1). Most patients had primary AML (*n* = 148, 59%); AML related to antecedent myelodysplastic syndrome (MDS) was present in 76 (30%) patients, and 27 (11%) had secondary AML related to treatment for a prior malignancy. The most common comorbidities were diabetes in 37 patients (15%) and coronary artery disease in 31 (12%) (Table 1).

Of the 251 patients, 157 (63%) received only one cycle of induction chemotherapy for active disease within one year, and 94 (37%) received ≥ 2 cycles, including 15 patients who received three cycles and one patient who received four cycles. A total of 75 (30%) patients received an allogeneic HCT, and 52 of the 75 (69%) developed either acute or chronic GVHD. The mean length of follow up from the onset of induction chemotherapy was 236 ± 140 days.

### 3.2. Invasive Fungal Disease (IFD)

Among the 251 patients, 17 patients (7%) had a proven (*n* = 4) or probable (*n* = 13) IFD. Sixteen episodes of possible IFD occurred in 14 patients for whom the mycological criteria of proven or probable IFD were not met. With the exception of 3 patients who had pulmonary consolidation or cavitation, patients with possible IFD had only pulmonary nodules on CT scan. Five patients were too ill to undergo further diagnostic studies and died within 11 days from the diagnosis of possible IFD. Patients with possible IFD were excluded from further analysis.

Among the 17 cases of proven and probable IFD, 12 (71%) were caused by molds. The most common IFD was invasive pulmonary aspergillosis (*n* = 6), followed by mucormycosis (*n* = 3), fusariosis (*n* = 3), *Pneumocystis* pneumonia (*n* = 3), and invasive candidiasis (*n* = 2) (Table 2).

Patients with proven or probable IFD were significantly older and had a greater number of cumulative neutropenic days when compared with those without IFD, HR 1.046 (95% CI 1.002- 1.093), *p* = 0.04 and HR 1.038 (1.018–1.059), *p* = 0.0001, respectively (Table 3).

By cause-specific proportional hazards regression, the risk for IFD increased by 3.8% for each day of neutropenia per 100 days of follow up. The risk for development of IFD was increased significantly in patients who had relapsed/refractory AML, HR = 7.562 (2.585–22.123), *p* = 0.0002.

Among the 17 patients with proven or probable IFD, five were undergoing primary induction or consolidation therapy, five had relapsed/refractory leukemia, and only one was in remission (Table 2). Four patients had received an HCT, and two of these had relapsed leukemia after the HCT.

Nine of the 17 (53%) patients with proven and probable IFD were not receiving antifungal prophylaxis at the time of the IFD diagnosis. Two patients did not receive *Pneumocystis* prophylaxis despite having an indication for this. Two patients were involved in clinical trials of experimental agents for the treatment of AML and did not have antifungal prophylaxis prescribed because the trial protocol prohibited the use of these agents. One patient had numerous drug-drug interactions and was unable to tolerate any antifungal prophylaxis. Three patients were no longer neutropenic, and another patient was no longer receiving chemotherapy. Infections in these nine patients included invasive aspergillosis (*n* = 4), invasive candidiasis (*n* = 2), mucormycosis (*n* = 1), and *Pneumocystis* pneumonia (*n* = 2) (Table 2).

Eight of the 17 (47%) proven and probable IFD were B-IFD, including aspergillosis (*n* = 2), fusariosis (*n* = 3), mucormycosis (*n* = 2), and *Pneumocystis* pneumonia (*n* = 1) (Table 2). Three patients receiving fluconazole prophylaxis developed invasive aspergillosis (*n* = 1) and fusariosis (*n* = 2), two patients who developed mucormycosis were taking voriconazole. The serum trough concentration of posaconazole (1580 ng/mL) and isavuconazole (3 µg/mL) demonstrated appropriate exposure when measured prior to occurrence of *Fusarium* pneumonia and invasive pulmonary aspergillosis, respectively. Exploratory proportional hazards regression analysis of cumulative antifungal exposure and development of IFD showed no significant differences among patients with IFD when compared with those without IFD, HR = 0.999 (0.983–1.015), *p* = 0.9. Using the Wilcoxon rank sums test, cumulative exposure to any specific antifungal agent was not associated with the development of B-IFD, *p* = 0.26, but cumulative exposure to non-mold active fungal prophylaxis (fluconazole) was associated with development of B-IFD (*p* = 0.012).

### 3.3. Outcomes

**Figure 1:**
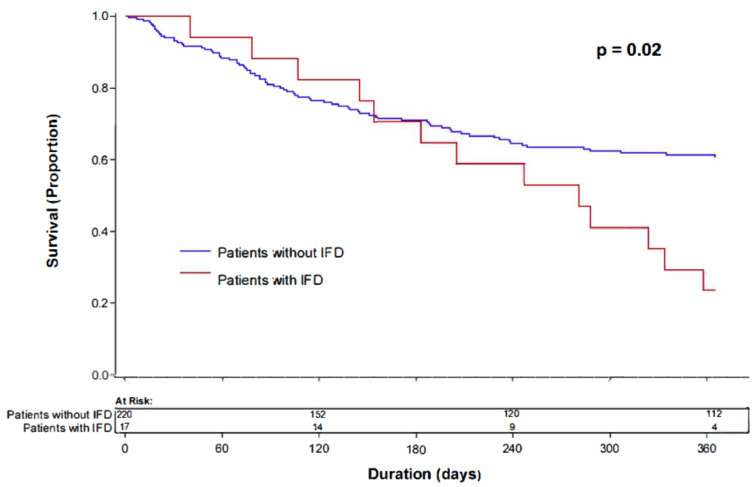
Kaplan-Meier analysis showing survival at one year after the date of first induction therapy for acute myeloid leukemia comparing patients who did or did not have invasive fungal disease.

Overall mortality in our cohort of 251 patients was 37% (*n* = 92) within the first year after receiving first induction chemotherapy for the treatment of AML. The mortality rate in patients who had relapsed/refractory AML was 70% (*n* = 77) compared with 12% (*n* = 15) in those without relapse, *p* < 0.001. Kaplan-Meier survival analysis showed significantly higher mortality at 1 year from first induction in patients who developed proven or probable IFD (13/17, 76%) when compared with those who did not develop IFD (79/220, 36%) (log-rank test, *p* = 0.02) (Figure 1).

Among the small number of patients who did not have relapsed/refractory AML, development of IFD was significantly associated with a higher mortality rate, *p* < 0.001. In those patients who had relapsed or refractory AML, no differences were noted among patients who developed IFD and those who did not, *p* = 0.26. All 13 patients with IFD who died did so within 12 weeks from the date of IFD diagnosis. Seven of nine patients (78%) with non-B-IFD and six of eight (75%) who had B-IFD died.

## 4. Discussion

Within a 1-year period from the time of initiation of induction therapy, 12% of patients with AML developed proven, probable or possible IFD. Of 17 patients (7%) who had proven or probable IFD, 8 (3%) were B-IFD in patients receiving antifungal prophylaxis. These rates are comparable with those reported from other centers [7,8,9,17,18] and slightly lower than we noted at our institution in the years 2010–2013 [10]. Mold infections accounted for 71% of proven and probable IFD, with *Aspergillus* the predominant organism, similar to prior reports [1,10]. Increasingly, however, non-*Aspergillus* molds are responsible for infections in patients with acute leukemia [19,20,21]. These more difficult-to-treat mold infections, including fusariosis and mucormycosis, have been reported more often in patients who were receiving antifungal prophylaxis, as we observed in our cohort. However, we did not isolate unusual and rare molds, such as *Scopulariopsis*, *Lomentospora*, *Lichtheimia*, and *Trichosporon*, as has been noted in other recent reports [8,19,20,21]. It is likely that geographic and environmental factors are as important as the specific antifungal agent used for prophylaxis to explain differences in the molds that predominate in various different institutions.

Cumulative duration of profound neutropenia was a significant risk factor for development of IFD among patients receiving chemotherapy for the treatment of newly diagnosed AML. We used a proportional hazard regression model and long-term follow up to determine the contribution of neutropenia to risk for IFD. We found a 3.8% increased risk of developing IFD by each additional day of neutropenia per 100 days follow up. Other studies have utilized the D-index, based on the area over the neutrophil curve which is defined by the duration and severity of neutropenia, as a predictor of risk factor for invasive mold infections in leukemia patients [22,23,24]. Our calculation differs in that it includes the cumulative duration of neutropenia over the first year, rather than depth and persistence of neutropenia during a single neutropenic episode prior to the diagnosis of IFD. Our study confirms findings from prior studies that neutropenia is a key risk factor for IFD and breakthrough IFD in patients with AML [5,6,10,11,25].

Developing an IFD had a significant impact on 1-year-survival, and this was most notable among patients whose leukemia was controlled. Aggressive chemotherapy allowing control of the leukemia likely contributed to the net state of immunosuppression and increased the risk for development of an IFD. For those patients who had relapsed/refractory disease, it appeared that both IFD and uncontrolled AML contributed to their poor outcomes.

GVHD is a known risk factor for the development of IFD. However, in our cohort, GVHD was not associated with an increased risk for IFD. This finding could be explained by the small number of patients who underwent HCT and developed GVHD (14%) and by the use of antimould prophylaxis in patients with GVHD.

Typically, antifungal prophylaxis for AML patients undergoing chemotherapy is initiated on the first day of neutropenia and continued until neutrophils are >500/µL. In our study, cumulative days of neutropenia and chemotherapy failure significantly increased the risk of IFD. These findings suggest that factors contributing to the net state of immunosuppression, rather than only the absolute neutrophil count, play a central role in the development of IFD. Novel approaches to antifungal prophylaxis perhaps should be devised to include alternative endpoints given that recovery of neutropenia alone is not wholly adequate to define decreased risk for IFD.

Current evidence supports the use of mould active prophylaxis for patients at high risk for IFD, such as those receiving induction chemotherapy for AML. Results from a large open label trial support the use of posaconazole to prevent IFD, and this drug is licensed for this indication [2]. There are not large studies of voriconazole prophylaxis in AML populations and the drug is not licensed for this indication, but effectiveness of this agent can be inferred from studies of the pre-engraftment neutropenic phase in HCT patients [26]. Furthermore, decisions about specific agents for antifungal prophylaxis in this patient population must take into account factors, such as the local epidemiology, especially the local incidence of Mucorales and other resistant moulds, drug interactions with chemotherapy agents, and costs.

In our study, 14 patients had 16 possible IFD and were excluded from further analysis; this constitutes almost 50% of all episodes of IFD, and 87.5% occurred despite antifungal prophylaxis. Similarly, in a prior study, almost 70% of episodes were designated as possible and were excluded from further analysis [10]. Mortality among patients with possible IFD was similar to that observed in patients with proven/probable IFD (73% and 76%, respectively). The lack of mycological evidence to support the diagnosis of IFD in this patient population may be secondary to the low yield of cultures, especially in the setting of widespread use of prophylaxis, limitations of non-culture methods, and an inability to use invasive methods in these patients to obtain tissue for culture and histopathology [27,28,29]. Possible IFD pose a dilemma for both clinicians and researchers. In clinical practice, patients with possible IFD typically receive empiric antifungal therapy, but treatment endpoints are unclear. Excluding this group of patients from analysis in clinical trials results in a decreased number of evaluable patients. Further understanding the impact and outcomes of possible IFD could lead to an improvement in the management of these patients.

The strengths of this study are related to the relatively large sample size, the homogenous patient population, and the use of recently updated definitions of IFD and B-IFD.

Our study has several limitations, including its retrospective and single-center design. We have excluded patients with episodes of possible IFD, which accounted for almost half of all episodes of IFD within a year of AML diagnosis, and excluding those episodes might have resulted in an underrepresentation of the real impact of IFD in this patient population. Finally, while the overall rate of IFD was similar to that reported in other studies, the actual number of patients with B-IFD was low, precluding further analysis or meaningful conclusions regarding risk factors for specific IFD in this population.

In conclusion, IFD remains an important problem among patients with AML despite the use of antifungal prophylaxis. Increasing cumulative days of neutropenia, as well as relapsed/refractory AML, correlate with increased risk of developing IFD, and development of IFD significantly increases mortality among patients with AML.

## Figures and Tables

**Table 1:** Characteristics of 251 patients undergoing first induction chemotherapy for acute myeloid leukemia.

Variable	*n* (%)
Male	138 (55)
Female	113 (45)
Age, years (mean ± std dev)	61.8 ± 14
**Hematologic malignancy**	
Primary AML	148 (59)
MDS with transformation to AML	76 (30)
Therapy-related AML	27 (11)
**Comorbid conditions**	
Diabetes mellitus	37 (15)
Coronary artery disease	31 (12)
Chronic obstructive pulmonary disease	24 (10)
Congestive heart failure	22 (9)
Rheumatologic disease	11 (4)
Interstitial lung disease	7 (3)
Heart transplant	2 (1)
**Induction chemotherapy regimens (no.)**	
1	157 (63)
≥2	94 (37)
**Allogeneic hematopoietic cell transplant**	75 (30)
Matched related	27
Matched unrelated	43
Haploidentical	4
Mismatched	1
**Graft-versus-host disease treatment**	52
Corticosteroids *	37
Tacrolimus	16
Anti-thymocyte globulin	4
Methotrexate	3

AML: acute myeloid leukemia, MDS: myelodysplastic syndrome; * defined as ≥ 0.3 mg/kg/day of prednisone equivalent.

**Table 2:** Proven or probable invasive fungal disease in 17 patients with acute myeloid leukemia.

Organism	Type of IFD	Site of IFD	Prophylaxis Agent	Timing of IFD Diagnosis (Days) ^1^	Status of AML at Time of IFD Diagnosis	Outcome at 12 Weeks
**Breakthrough IFD**						
*Aspergillus* (*n* = 2)	Proven	Pleural space ^2^	Fluconazole	81	Primary consolidation	Died
	Probable	Pulmonary ^3^	Isavuconazole	321	Post-allo HCT	Died
*Fusarium* (*n* = 3)	Proven	Disseminated	Fluconazole	242	Relapsed refractory	Died
	Probable	Pulmonary	Fluconazole	358	Relapsed after allo HCT	Survived
	Probable	Pulmonary	Posaconazole	320	Relapsed after allo HCT	Died
*Mucorales* (*n* = 2)	Proven	Disseminated	Voriconazole	150	Relapsed refractory	Died
	Probable	Pulmonary	Voriconazole	29	Primary induction	Died
*Pneumocystis* (*n* = 1)	Probable	Pulmonary	Pentamidine (inh)	15	Primary induction	Survived
**Non-Breakthrough IFD**						
*Aspergillus* (*n* = 4)	Probable	Pulmonary ^3^	--	262	Relapsed refractory	Survived
	Probable	Pulmonary ^3^	--	48	Primary induction	Survived
	Probable	Pulmonary ^3^	--	256	Relapsed refractory	Died
	Probable	Pulmonary ^3^	--	137	Primary consolidation	Died
*Candida* (*n* = 2)	Probable	Invasive candidiasis	--	261	Remission	Died
	Proven	Invasive candidiasis	--	340	Initial relapse	Died
*Pneumocystis* (*n* = 2)	Probable	Pulmonary	--	22	Primary refractory	Died
	Probable	Pulmonary	--	197	Post-allo HCT	Died
*Mucorales* (*n* = 1)	Probable	Pulmonary	--	365	Relapsed refractory	Died

AML: acute myeloid leukemia; Allo HCT: allogeneic hematopoietic cell transplant; IFD: invasive fungal disease; inh: inhaled. ^1^ Timing of IFD diagnosis from study entry date (in days). ^2^ positive pleural fluid culture for *Aspergillus flavus.* ^3^ Probable invasive pulmonary aspergillosis determined on basis of positive serum galactomannan in 3 patients (range 1.2 –3.75 ODI), positive BAL galactomannan in 2 (range 0.9–2.3 ODI).

**Table 3:** Univariable analysis of risk factors for the development of proven/probable invasive fungal disease (IFD) in patients with acute myeloid leukemia ^1^.

Risk Factor	No IFD *n* = 220	IFD *n* = 17	Hazard Ratio (95%CI)	*p* Value
Age, years (mean ± std dev)	61 ± 15	66 ± 12	**1.046 (1.002–1.093)**	**0.04**
Gender				
Male	119	11	0.588 (0.218–1.591)	0.30
Female	101	6		
Hematological disease				
Primary AML	132	12	1.062 (0.37–3.05)	0.91
MDS with transformation to AML	62	5	0.52 (0.18–1.499)	0.23
Therapy-related AML	26	0		
Cycles of induction chemotherapy				
1	143	10	0.885 (0.337–2.326)	0.80
≥2	77	7		
AML status (relapse/refractory)	99	12	**7.562 (2.585–22.123)**	**0.0002**
Allogeneic hematopoietic cell transplant	68	4	2.638 (0.854–8.149)	0.09
Graft vs. host disease	47	3	2.047 (0.586–7.15)	0.26
Cumulative days of neutropenia (mean ± std dev)	28 ± 25	34 ± 20	**1.038 (1.018–1.059)**	**0.0001**

AML: acute myeloid leukemia, MDS: myelodysplastic syndrome. ^1^ Patients with possible IFD were excluded from the analysis.
